# Task-based quantification of image quality using a model observer in abdominal CT: a multicentre study

**DOI:** 10.1007/s00330-018-5518-8

**Published:** 2018-06-01

**Authors:** Damien Racine, Nick Ryckx, Alexandre Ba, Fabio Becce, Anais Viry, Francis R. Verdun, Sabine Schmidt

**Affiliations:** 1 Institute of Radiation Physics, Lausanne University Hospital, Rue du Grand-Pré 1, 1007 Lausanne, Switzerland; 2 Department of Diagnostic and Interventional Radiology, Lausanne University Hospital, Rue du Bugnon 46, 1011 Lausanne, Switzerland

**Keywords:** Abdominal computed tomography, Image quality, Model observer, Standardisation, Task-based assessment

## Abstract

**Objective:**

We investigated the variability in diagnostic information inherent in computed tomography (CT) images acquired at 68 different CT units, with the selected acquisition protocols aiming to answer the same clinical question.

**Methods:**

An anthropomorphic abdominal phantom with two optional rings was scanned on 68 CT systems from 62 centres using the local clinical acquisition parameters of the portal venous phase for the detection of focal liver lesions. Low-contrast detectability (LCD) was assessed objectively with channelised Hotelling observer (CHO) using the receiver operating characteristic (ROC) paradigm. For each lesion size, the area under the ROC curve (AUC) was calculated and considered as a figure of merit. The volume computed tomography dose index (CTDI_vol_) was used to indicate radiation dose exposure.

**Results:**

The median CTDI_vol_ used was 5.8 mGy, 10.5 mGy and 16.3 mGy for the small, medium and large phantoms, respectively. The median AUC obtained from clinical CT protocols was 0.96, 0.90 and 0.83 for the small, medium and large phantoms, respectively.

**Conclusions:**

Our study used a model observer to highlight the difference in image quality levels when dealing with the same clinical question. This difference was important and increased with growing phantom size, which generated large variations in patient exposure. In the end, a standardisation initiative may be launched to ensure comparable diagnostic information for well-defined clinical questions. The image quality requirements, related to the clinical question to be answered, should be the starting point of patient dose optimisation.

**Key Points:**

• *Model observers enable to assess image quality objectively based on clinical tasks.*

• *Objective image quality assessment should always include several patient sizes.*

• *Clinical diagnostic image quality should be the starting point for patient dose optimisation.*

• *Dose optimisation by applying DRLs only is insufficient for ensuring clinical requirements.*

## Introduction

In diagnostic radiology, computed tomography (CT) contributes to a major part of the public radiation dose exposure, which leads to public concern over potential cancer induction risks [1–4]. Many initiatives have been launched to avoid unnecessary or useless exposure, such as Image Gently and Image Wisely. The introduction of diagnostic reference levels (DRLs) allowed, to a certain extent, a reduction in the heterogeneity of the delivered dose exposure from one institution to another [5]. However, the DRLs provided for CT examinations are generally defined as a function of an anatomical region, which is certainly a limitation, as a given anatomical region may not need the same image quality depending on the specific clinical question (e.g., head trauma vs. ischaemic stroke). In addition, technological developments, such as the automatic tube current modulation (ATCM), using dynamic beam collimation with less over-ranging have been proposed to drastically reduce patient exposure [6]. Furthermore, in the last 10 years, iterative reconstruction (IR) techniques have become increasingly popular as a mechanism to reduce CT dose exposure while ensuring image quality. In general, IR techniques allow drastic noise reductions while maintaining a reasonable spatial resolution compared to traditional filtered back-projection (FBP) techniques [7–12].

With the large number of IR solutions now proposed by multiple CT vendors, it has become crucial to systematically evaluate the dose reduction potential and subsequent image quality for each technique. These investigations have been performed using both clinical images and phantom images [13–15]. Despite the production of subjectively better looking images, IR techniques do not allow full recovery of the detection of low-contrast structures when the applied dose reductions are too high [16, 17]. Thus, when dealing with dose reductions by means of IR, the low-contrast detectability (LCD) should systematically be investigated using task-based image quality assessment methodologies. Given the large number of IR solutions proposed by CT manufacturers, such image quality assessments should be performed using phantoms and objective quantitative methods. The US Food and Drug Administration recommends the use of mathematical model observers as surrogates to human observers [18]. The outcomes provide image quality metrics measured on phantoms as image quality indicators, while the volume CT dose index (CTDI_vol_) or the dose length product (DLP) are used as patient exposure indicators.

The aim of the present study was to investigate the variability of patient exposure and the CT image quality provided by a large number of centres when evaluating the presence or absence of focal abdominal lesions in phantoms of different sizes.

## Materials and methods

### Abdomen phantom and image acquisition

**Fig. 1:**
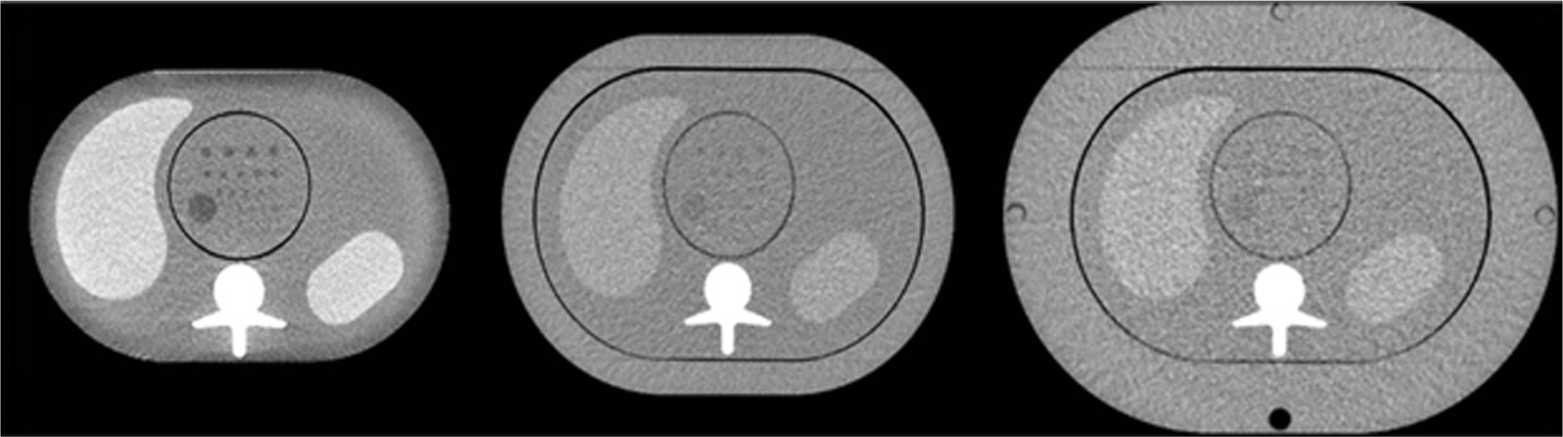
CT images of the anthropomorphic abdomen phantom. From left to right: small, medium and large phantom

An anthropomorphic abdomen phantom (QRM 401) simulating three types of attenuations produced by an adult patient (Fig. 1) was used for this study. This phantom was called the small phantom (anterior posterior (AP) x lateral diameter: 20 x 30 cm) and represented a thin adult with a body mass index (BMI) of 20 kg/m^2^ or a patient weight of ~50 kg. To vary the patient’s morphology, two additional rings, one medium-sized (2.5 cm in thickness) and one large-sized (5 cm in thickness), were added to the phantom. With these two extra rings (AP x lateral diameter: 25 x 35 cm, and 30 x 40cm, respectively) the phantom represented patients with a BMI of 26 kg/m^2^ (patient weight ~90 kg) and 35 kg/m^2^ (patient weight ~120 kg), respectively. A cylindrical module containing spherical lesions 8, 6 and 5 mm in diameter with a contrast of 20 HU relative to the background at 120 kVp was inserted in the centre of the phantom (four spheres per diameter). It is worth mentioning the phantom is made of various plastics that do not contain any high atomic number materials. Thus, the variation of contrast with x-ray beam energy is negligible.

The three abdomen phantom sizes were scanned on 68 CT scanners installed in 62 centres (Table 1). The four major manufacturers, GE Healthcare, Philips Healthcare, Siemens Healthineers and Canon Medical System, were represented. GE and Philips accounted for 68% of the CT units involved in this study. Each data acquisition was performed according to the local clinical acquisition and image reconstruction parameters of the portal venous phase. To maximise the performance of the automatic tube current modulation (ATCM) the phantoms were always positioned at the isocentre of the CT scanners [6, 19, 20]. To provide comparable spatial resolution, the scanned and reconstructed field of view were set to 320 mm, 370 mm and 420 mm for the small, medium and large phantoms, respectively. To ensure statistical robustness of the results, the phantom was scanned ten times on each CT unit without changing its position between the different acquisitions. This resulted in 40 images containing a signal and 90 images that contained only noise [19, 20].

**Table 1:** The 68 CT scanners involved in this study

	CT scanner	Number
GE	BrightSpeed S	1
	Discovery CT 750 HD	2
	LightSpeed VCT	7
	LightSpeed16	1
	Optima CT520	2
	Optima CT580	1
	Optima CT660	7
	Revolution	1
	Revolution EVO	2
Philips	Brilliance 40	1
	Brilliance 64	6
	iCT 256	4
	Ingenuity Core 128	4
	Ingenuity CT	4
	Ingenuity Flex	2
	IQon - Spectral CT	1
Siemens	Perspective	1
	Sensation 64	2
	SOMATOM Definition AS	2
	SOMATOM Force	1
Canon Medical System	Activion16	4
	Aquilion	6
	Aquilion PRIME	6

### Image quality assessment

A channelised Hotelling observer (CHO) with dense difference of Gaussian (DDoG) channels was used to assess the LCD. The chosen channels are known to represent the spatial selectivity behaviour of the human primary visual cortex (V1). With this channelisation process of the images (each image is passed through the DDoG channels) the model observer is considered anthropomorphic. In our study we used ten dense differences of Gaussian channels, since this value has been known to be sufficient for mimicking well the human detection in such a simple task [21–23].

Each channel is defined by Eq. 1:

1 $$ {C}_j\left(\rho \right)={e}^{-\frac{1}{2}{\left(\frac{\rho }{Q{\sigma}_j}\right)}^2}-{e}^{-\frac{1}{2}{\left(\frac{\rho }{\sigma_j}\right)}^2} $$

Where ρ was the spatial frequency, σ_j_ the standard deviation of each channel and Q the filter bandwidth. Each standard deviation of the j^th^ channel value was given by σ_j_ = σ_0_ α^j-1^. As previously described [21], the following variable settings were: σ_0_ = 0.005, α = 1.4 and Q = 1.67.

As in other CHO models, the CHO model with DDoG channels computes a decision variable, λ, from the dot product between a channelised image v and a template w, as seen in Eq. 2:

2 $$ {\lambda}_{n,s,i}={\mathbf{w}}^T{\mathbf{v}}_{n,s,i} $$

The decision variable can be seen as a grade given by the model to the images. The higher the decision variable, the higher the probability of the presence of a signal in the image. For this study, 90 signal-absent images were used to compute 90 decision variables for the signal-absent image category and 40 signal-present images were used to compute 40 decision variables for the signal-present image category. In Eq. 2, T is the transpose operator; n represents the image category, signal-absent or signal-present, s represents the lesion size, and i the image number. The template (w) takes into account the statistical knowledge of noise by computing the covariance matrix K from 90 channelised images containing no signal. The template w also takes into account the signal by computing a theoretical signal that represents the different lesions sizes, as seen in Eq. 3:

3 $$ \mathbf{w}={\left({\mathbf{K}}_{v/\mathrm{n}}\right)}^{-1}{\mathbf{v}}_{Theo} $$

Only the phantom sphere measuring 5 mm in diameter was used in this study, as preliminary measurements showed that this size was the most critical parameter in terms of area under the receiver operating characteristic (ROC) curve (AUC). To avoid the need for acquiring too many images, we used a theoretical signal of 5 mm in diameter (v_Theo_) instead of a mean signal (the mean signal is defined as the difference between the mean of images containing a signal and the mean of images that contained only noise) for the signal template. It was created using a simulated 2D Gaussian curve with a full width at half maximum of 5 mm. In addition, this method avoids overfitting of the data. Using the decision variable distribution for the signal-absent category (90 decision variables) and the signal-present category (40 decision variables), a ROC study was then computed and characterised by its the AUC. The latter was a surrogate to assess image quality. The average and standard deviation of the AUC were estimated using a bootstrap method [24]. In practice, the model performed 500 ROC experiments for each category. No internal noise was added to improve the match between the model observer and the human observer performance [25].

The displayed CTDI_vol_ was used as a figure of merit for the patient dose exposure. We did not systematically measure these displayed CTDI_vol_ values, since our national legislation requires a conformity check every 3 months. This conformity check must be performed by the manufacturer and ensures that the displayed and measured CTDI_vol_ values are equal with a permitted difference of ≤ 20%.

As the four major manufacturers were represented in this study, two different types of ATCM were involved in the data acquisitions: one ATCM for which the user had to choose a noise level (GE and Canon Medical System) and another ATCM for which the user only introduced a reference image load (in mAs, Philips and Siemens). To study the impact of the ATCM method on the image quality when the patient size varied, the correlation between the AUC of the different phantom sizes was calculated using the Pearson coefficient (r). Correlation is an effect size and so we can verbally describe the strength of the correlation using the guide that Evans et al. suggest for the absolute value of r [26]: 0.00–0.19 ‘very weak’, 0.20–0.39 ‘weak’, 0.40–0.59 ‘moderate’, 0.60–0.79 ‘strong’ and 0.80–1.0 ‘very strong’.

## Results

### CTDI_vol_ in terms of patient size

**Fig. 2:**
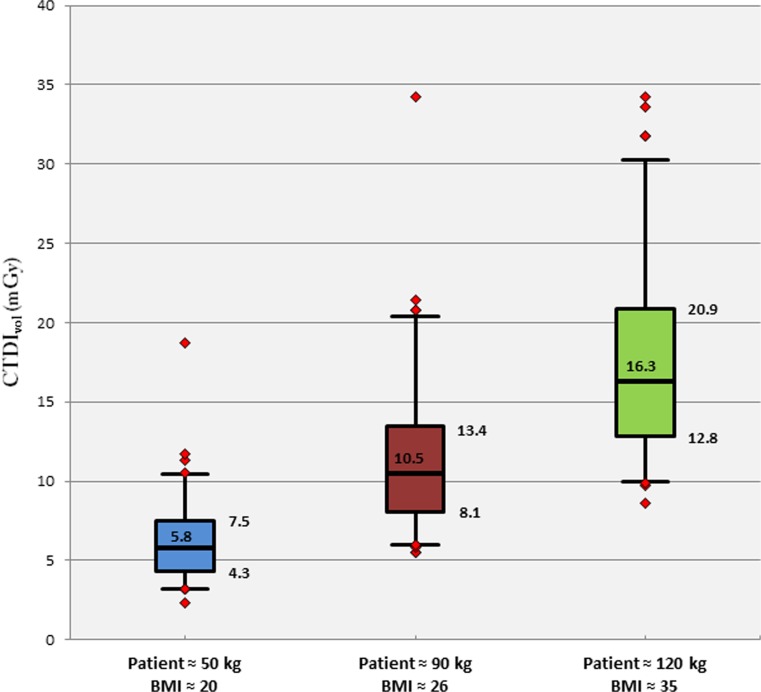
CTDI_vol_ obtained for the three phantom sizes as a function of the abdominal clinical CT protocol settings. The black line in the middle of the coloured rectangles represents the median. The bottom edge of the rectangle corresponds to the first quartile and the top edge to the third quartile. The bottom line represents the fifth percentile and the top line the 95th percentile. The red dots outside these two lines are outliers

As expected, the CTDI_vol_ increased with growing phantom size. Due to the selection of the locally implemented clinical CT protocol for each unit, the CTDI_vol_ varied significantly for a given phantom size. For the small phantom, the CTDI_vol_ varied from 2.3 to 18.7 mGy with a median of 5.8 mGy and third quartile of 7.5 mGy. For the medium-sized phantom, the CTDI_vol_ varied from 5.5 to 34.2 mGy with a median of 10.5 mGy and third quartile of 13.4 mGy. For the largest phantom, the CTDI_vol_ varied from 8.6 to 34.2 mGy with a median of 16.3 mGy and a third quartile of 20.9 mGy (Fig. 2).

### Image quality for abdomen CT protocols

#### Small phantom

**Fig. 3:**
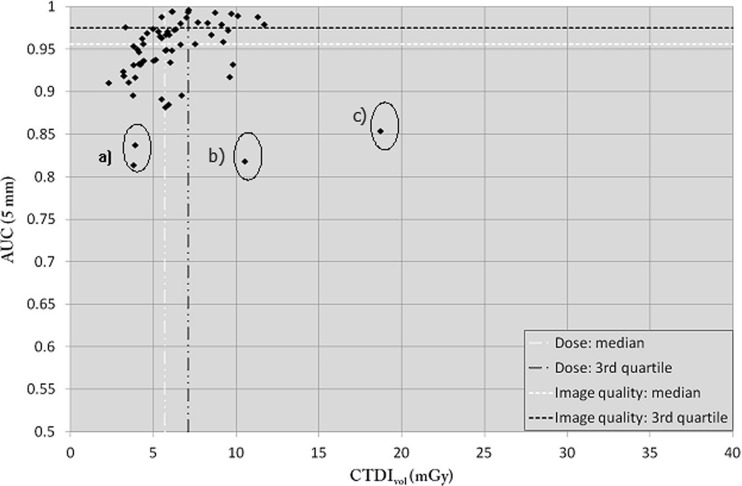
AUC as a function of CTDI_vol_ for the small-sized phantom

For the small phantom (Fig. 3), despite the use of relatively low CTDI_vol_ values (median CTDI_vol_ 5.8 mGy) compared to the national DRLs provided for abdominal CT protocols (15 mGy) [27], excellent image quality (AUC ≥0.95) was obtained for most of the centres (Fig. 3) [28]. Indeed, the median AUC was 0.96 and the third quartile was 0.97, with several centres achieving an AUC >0.99. Only three centres had an AUC <0.85. Two protocols (centre a, see Fig. 3) used too low a dose level for the CT and therefore only achieved an image quality with an AUC inferior to 0.85. The slice thickness used for centres b and c was equal to 5 mm and was thus not compatible for accurately detecting a lesion 5 mm in diameter due to partial volume effect, even if the dose was high.

#### Medium-sized phantom

**Fig. 4:**
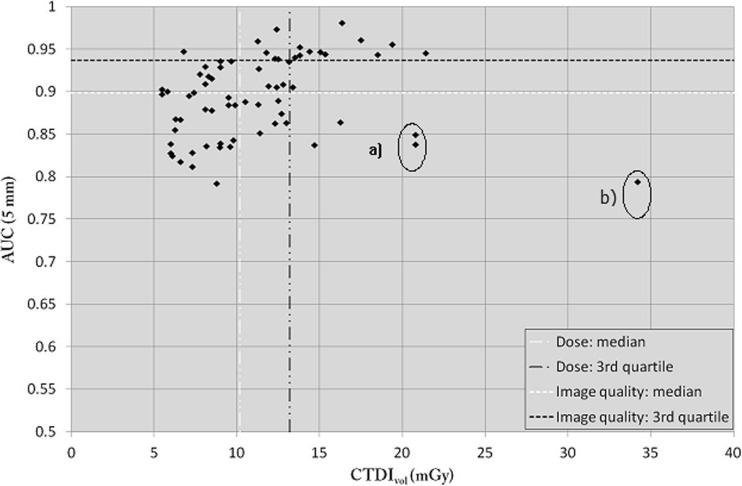
AUC as a function of CTDI_vol_ for the medium-sized phantom

For the medium-sized phantom (Fig. 4), the median CTDI_vol_ was 10.2 mGy with a median AUC of 0.90 (third quartile 0.94). With this phantom size, almost a quarter of the centres had an AUC <0.85. The dose levels were relatively or significantly high in three protocols (centres a and b, see Fig. 4) due to a suboptimal reconstruction slice thickness of 5 mm. These images were obtained on the oldest CT scanners included in this study, which had been introduced on the market in 2007–2009.

#### Large phantom

**Fig. 5:**
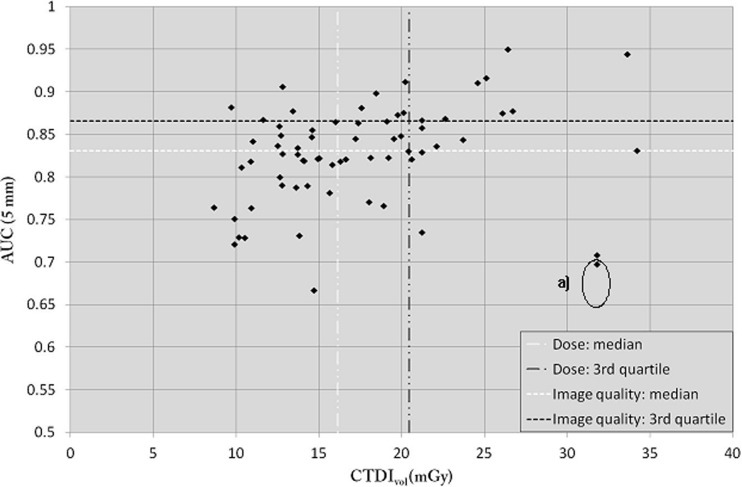
AUC as a function of CTDI_vol_ for the large-sized phantom

For the large phantom (Fig. 5), the median CTDI_vol_ was 16.1 mGy, with a median AUC of 0.83 (third quartile 0.87). With this phantom size, almost a quarter of the centres had an AUC <0.80. As with the other phantom sizes, the image quality was significantly low in two protocols (centre a, see Fig. 5) due to partial volume effects associated with the use of one of the oldest CT units.

#### Image quality correlation

For the first type of ATCM for which the user had to choose a noise level, the correlation (r) of AUC values between the different phantom sizes varied from 0.33 between small and large to 0.49 between medium and large (Table 2). For the second type of ATCM, for which the user only introduced a reference image load (in mAs), the correlation (r) of AUC values between the different phantom sizes varied from 0.40 between small and large to 0.58 between medium and large (Table 3). Thus, there was a weak to moderate positive correlation between the level of CT image quality and phantom size.

**Table 2:** Correlation matrix based on the area under the ROC curve (AUC) obtained with automatic tube current modulation (ATCM) that maintained absolute noise levels close to target values

Size	S	M	L
S	1		
M	0.39	1	
L	0.33	0.49	1

**Table 3:** Correlation matrix based on the area under the ROC curve (AUC) obtained with automatic tube current modulation (ATCM) that maintained a constant level of overall diagnostic quality for all patient sizes relating to a reference image

Size	S	M	L
S	1		
M	0.49	1	
L	0.40	0.58	1

## Discussion

To justify a given CT examination, the imaging protocol should be adapted to answer one or several specific clinical questions corresponding to the indication of the examination. In this study, LCD was assessed using the portal venous phase for the detection of focal liver lesions. We thus created a task-based approach by means of an anthropomorphic CHO model observer applied on three abdominal phantom sizes. Using this methodology, we showed that some clinical protocols applied by several CT units across the country allowed production of a high image quality level in a low-dose range. Nevertheless, some protocols applied on similar or different CT units produced lower AUC levels despite the use of a comparable dose range. This was sometimes due to the use of sub-optimal ATCM settings or sub-optimal reconstructed slice thicknesses (Figs. 4 and 5). Further investigations are needed to fully understand if a better outcome could be obtained from the CT units that used high dose levels.

Taylor et al. have recently shown that samples of approximately 300 CT examinations of patients with a body weight in the range of 67–73 kg are necessary to create reliable DRLs [29]. For many years, broad application of the DRL concept has allowed national homogenisation of patient exposure in practice for specific anatomical regions [30, 31]. In our study, it appears that the image quality obtained using a task-based image quality approach was relatively homogeneous among all centres with the small phantom; yet when scanning larger phantom sizes, the image quality tended to vary between the different centres. However, for a given clinical question, there is large variability in dose especially when dealing with large patients, due to different ATCM settings. Therefore, it is important to use different phantom sizes to assess the behaviour of image quality when varying the exposure and patient size. All these variations in terms of dose and patient exposure provide a weak-to-moderate correlation between the outcomes of image quality when varying the phantom sizes (Tables 2 and 3). Indeed, the local practice (especially the setting of the maximum mA during the image acquisition) and/or the limitation of the x-ray tube power influences the feasibility of using an equivalent image quality level for each patient’s morphology. In such cases, the acquisition parameters had to be adjusted accordingly, but the optimisation of acquisition parameters and patient dose was not reached in most of the centres included in our study. When the national/international DRLs are used to optimise the clinical CT protocols, image quality is currently not considered. Our study demonstrates that the DRL concept, when dealing with patient-dose optimisation, has reached its limits since comparable dose levels applied on different CT units provided highly variable diagnostic image quality levels. The optimisation of clinical CT protocols should rather ensure that a comparable diagnostic information, for well-defined clinical questions, is obtained at the lowest achievable dose on different CT units. Trying to only reach comparable patient-dose exposures on different CT units has a limited potential in the framework of patient exposure and image quality optimisation.

Therefore, in the end, the challenge is to establish a link between the different clinical tasks and the surrogates used to assess image quality [32]. These task-based image quality criteria (for example the LCD requirements) should be initiated for a few morphology types and a standardisation process concerning image quality requirements as a function of the common clinical indications [33]. Indeed, IR provide images that look satisfying in a larger dose range than with the standard FBP reconstruction algorithm, since the amount of noise does not alert the radiologist. However, ‘blind’ dose reductions could be done that may impair the diagnostic image quality. Therefore one should ensure the presence of the necessary amount of information by objectively and quantitatively evaluating image quality to fully benefit from the potential of dose reductions provided by the use of IR [34].

This study has limitations. First, we did not consider intravenous contrast medium administration, which was an important part of the optimisation protocol and has an undeniable impact on contrast enhancement of organs and vessels and, thus, on the diagnostic yield; yet this method enables up to 50% dose reduction [35]. Second, due to the use of a homogeneous phantom that simplifies the objective assessment of image quality, it is far from the current clinical situation with heterogeneous backgrounds. However, we still consider this as a good starting point for the optimisation process. Third, the largest phantom was made of muscle tissue only, which is not fully representative of obese patients. This increased the performance requirements for the investigated CT units. The largest rings added to the core of the phantom should ideally have contained both muscle tissue and fat. Four, the material composing the lesion inserted inside the phantom would have been more clinically relevant if it contained a high Z material, such as iodine, when we investigated the portal venous phase in an abdominal CT protocol, a de facto injected phase. Finally, several other data acquisition and image reconstruction parameters, such as ATCM, tube voltage, slice thickness and IR level, influence the overall CT image quality. However, our primary aim was rather to highlight the wide spread of image quality and dose for a single clinical task. Assessing the confounding effects of all these parameters separately would require further dedicated studies and was beyond the scope of the present investigation.

In summary, we developed an objective, quantitative and robust method for benchmarking abdominal CT protocols using three different phantom sizes and evaluating 68 different CT units from 62 centres across the country. Our study demonstrates that radiologists, radiographers and medical physicists, must collaborate to ensure that dose reductions do not lead to sub-optimal images, which possibly impair diagnostic quality. The aim of the previously introduced DRL concept was to reduce the variability of patients’ dose exposure, but as we have shown here, it is insufficient to ensure comparable image quality on different CT machines. We should now define a set of task-based image quality criteria related to well-defined clinical indications and work towards the standardisation of image quality requirements. To establish these requirements, it is important to define the critical target to be detected and determine the AUC level to be used for standardised phantoms.

## Abbreviations

ATCM: Automatic tube current modulation
AUC: Area under the ROC curve
BMI: Body mass index
CHO: Channelised Hotelling observer
CT: Computed tomography
CTDI_vol_: Volume computed tomography dose index
DDoG: Dense difference of Gaussian
DLP: Dose length product
DRL: Diagnostic reference level
FBP: Filtered back-projection
IR: Iterative reconstruction
LCD: Low-contrast detectability
ROC: Receiver operating characteristic
